# Protective effect of tomato pomace extract encapsulated in combination with probiotics against indomethacin induced enterocolitis

**DOI:** 10.1038/s41598-024-52642-y

**Published:** 2024-01-27

**Authors:** Karem Fouda, Ahmed M. Mabrouk, Sherein S. Abdelgayed, Rasha S. Mohamed

**Affiliations:** 1 Nutrition and Food Sciences Department, National Research Centre, Dokki, Cairo, Egypt; 2 Dairy Department, National Research Centre, Dokki, Cairo, Egypt; 3 Pathology Department, Faculty of Veterinary Medicine, Cairo University, Cairo, Egypt

**Keywords:** Biochemistry, Health care

## Abstract

Tomato pomace (TP), an antioxidant-rich byproduct, may be suitable for noble applications. The regulation of ROS generation and the anti-inflammatory response can help to prevent ulceration. The purpose of this study was to examine TP for antioxidants, in silico anti-inflammatory properties, and its potential to protect against ulceration and erosion triggered by indomethacin. Tomato pomace extract (TPE) was encapsulated either alone or with probiotics to maximize its potential effect. These microcapsules were investigated in indomethacin-treated rats. TPE demonstrated antioxidant activity as well as high levels of carotenoids (15 mg/g extract) and polyphenols. Because of their binding affinity as well as hydrophobic and hydrogen bond interactions with the active sites of TNF-α and IL-1β inflammatory cytokines, ellagic acid and rutin may be implicated in the anti-inflammatory effect of TPE, according to the docking study. TPE microcapsules, either alone or in combination with probiotics, demonstrated a protective effect against enterocolitis by reducing oxidative stress and inflammation, as evidenced by the decrease in stomach and intestinal MDA, NO, IL-1β, IL-6, and TNF-α levels and the increase in CAT, SOD, and GSH activities. The produced microcapsules are suggested to be promising candidates for protection against gastric ulcers and erosion.

## Introduction

Tomato pomace (TP) is a by-product rich in nutrients and bioactive components such as phenolic compounds, carotenoids (lycopene, carotenes, and lutein), sugars, and fibers. Therefore, it may be suitable for noble applications. The advantages of valorizing TP include reducing food waste, minimizing environmental disruption, and generating products with added value for food and pharmaceutical applications. The bioactive components of TP provide health benefits due to several biological effects such as antioxidant, anti-hypertensive, anti-proliferative, and anti-inflammatory^1^. Polyphenols, the most widespread phytochemicals, have been demonstrated to have apoptotic, immunomodulatory, anti-inflammatory, and antioxidant activities. In addition to locally reducing oxidative stress, their actions can be directed at cellular targets by changing the expression of genes linked to inflammation, such as NF-B, Nrf-2, Jak/STAT, and MAPKs, inhibiting the production of downstream cytokines (such as IL-8, IL-1β, and TNF-α), and enhancing the body’s antioxidant enzymes (SOD and GPx). Furthermore, as prebiotics, they may be implicated in probiotic viability improvement, healthy microbiota maintenance, short-chain fatty acid production, gut permeability enhancement, and tight junction stability improvement^2^.

Probiotics are living bacteria that, when given in appropriate amounts, alter the gut microbiota. Because probiotics help to restore the mucosa and promote anti-inflammatory effects, they considerably reduce intestinal inflammation when used as a treatment, eliminate discomfort and edema, and overall improve the quality of life. It’s also important to point out that probiotics are significantly less expensive and have less potential side effects than other forms of gastrointestinal inflammation treatment^3^. Combining probiotic strains with phenol-rich extract as antioxidants may be beneficial in extending their viability and providing health benefits to the host^4^.

Several studies have demonstrated that increased reactive oxygen species (ROS), decreased cell proliferation, and increased inflammation can all lead to stomach injury. It is critical to regulate ROS generation and the anti-inflammatory response to prevent stomach ulcers^5^. Free radicals of reactive oxygen (and nitrogen) are also involved in the pathophysiology of inflammatory bowel disease (IBD), which includes Crohn’s disease (CD), which affects all portions of the digestive system, and ulcerative colitis (UC), which affects the large intestine. Protective agents and alternative treatments for gastrointestinal inflammation may be beneficial in preventing the side effects of the conventional treatments (antibiotics, corticosteroids, immunosuppressants, and inhibitors of tumor necrosis factor), which include hemopathies, diarrhea, vomiting, and thrombocytopenia^6^.

Tomato pomace may be potentially useful for the treatment and prevention of gastrointestinal inflammation^7^ due to its bioactive components, notably carotenoids and phenolic compounds, which have powerful antioxidant and oxygen-quenching activities. Nutritional regimens supplemented with bioactive substances may help to create an environment ideal for the growth of beneficial bacteria, treat intestinal dysbiosis, prevent recurrence, and promote full recovery from gastrointestinal inflammation. However, in the absence of concurrent probiotic therapy, these nutritional regimens may be ineffective^8^. To sustain the desired advantages, probiotics and bioactive compounds need to be protected. Microencapsulation has been considered a successful strategy in the food industry for the development of functional foods. It improves the handling and utilization of the bioactive compounds, controls the release of the bioactive compounds on demand and to specific targets, and increases the bio-availability of the bioactive compounds^1^.

This study was designed to achieve several goals. The first was an evaluation of the characteristics and in silico anti-inflammatory effect of tomato pomace extract. The second was producing microcapsules from tomato pomace extract, either alone or in combination with probiotics. The third was to assess the protective effect of the produced microcapsules against indomethacin-induced enterocolitis in a rat model.

## Materials and methods

### Materials

Tomato fruits (*Solanum lycopersicum*) were purchased from an outlet selling Egyptian Ministry of Agriculture products in Giza Governorate, Egypt. In this study, tomatoes grown in Egyptian Ministry of Agriculture farms and harvested in October 2022 were used to be identical in cultivation. Probiotics, *Lactobacillus acidophilus* (LA-5) and *Bifidobacterium bifidum* (Bb-12)*,* were obtained from Chr. Hansen`s Laboratory, Copenhagen, Denmark. Sodium alginate (medium viscosity) was purchased from Loba Chemie, Pvt Ltd—Mumbai, India. 2,2-Dipheny l-1-picryl hydrazyl (DPPH) and Trolox were purchased from Sigma-Aldrich Chemical Co., (St. Louis, USA). All of the solvents, chemicals, and culture media used in this study are of analytical grade.

### Methods

#### Preparation of tomato pomace extract (TPE)

Tomato fruits were washed, cut, and squeezed out with a manual tomato juicer. The pomace (mix of seeds and peels) was freeze-dried at − 47 °C and 0.100 mbar for 48 h, fine-grained, sieved, and then kept at 4 °C for further use. According to Solaberrieta et al.^9^, ultrasound-assisted extraction (UAE) was used to prepare the tomato pomace extract. In a 150 ml beaker, freeze-dried tomato pomace (2.0 g) was mixed with 100 ml of ethanol. The ultrasonic probe was submerged in the beaker, and it was chilled in an ice bath. The extraction employed ethanol: water (60% v/v), a 10-min extraction time, and a 70% amplitude as optimal conditions for obtaining the highest yield, according to the results of Solaberrieta et al.^9^. Tomato pomace extract was centrifuged for 10 min at 5300 rpm. The extraction solvent was used to wash the solid residue twice and then discarded. Subsequently, ethanol was evaporated under decreased pressure (around 75 torr, 99 mbar at 55 °C), and water was removed using a freeze dryer (Crest Alpha 1–4 LSC plus Germany) at − 47 °C and 0.100 mbar for 48 h. The extraction yield was measured by using the following equation:

1 $${\text{Yield }}\left( \% \right) \, = {\text{ TPE}}/{\text{TP}}*{1}00$$

where TPE is the weight of extract obtained after freeze-drying, and TP is the weight of dried tomato pomace.

#### Estimation of total phenolic content of TPE

The total phenolic content of TPE was measured using the Folin-Ciocalteu test, as modified by Toor and Savage^10^. An aliquot (0.5 ml) of the extract was mixed with 2.5 ml of Folin-Ciocalteu reagent that had been previously diluted in distilled water (1:10, v/v). Aqueous sodium carbonate (2.0 ml of 7.5 wt%) was added. The mixture was then vortexed, and the absorbance was measured at 765 nm after 30 min of incubation at 45 °C in the dark. Gallic acid in ethanol: water (60% v/v) was employed as a quantitative reference standard. The results were given in milligrams of gallic acid equivalents (GAE) per gram of dried TP. The extract was estimated in triplicate.

#### Estimation of antioxidant Activity of TPE

TPE’s DPPH scavenging activity was assessed using the method of Szabo et al.^11^. TPE (0.4 ml) was mixed with 2.1 ml of newly prepared DPPH solution (10^−4^ mol L^−1^ in ethanol). The mixture was vortexed and incubated in the dark at room temperature for 120 min. The absorbance was measured at 517 nm in comparison to a pure ethanol blank. Trolox in ethanol: water (60% v/v) was employed as a quantitative reference standard. The results are given in milligrams of Trolox equivalents (TE) per gram of dried TP. The extract was estimated in triplicate.

#### Estimation of total carotenoid content of TPE

Total carotenoid concentration was spectrophotometry measured using the method of Jamaleddine et al.^12^. TPE (0.1 g) was ultrasonicated for 1 min in 50 ml ethanol. Total carotenoid content was calculated by measuring the absorbance at 446 nm and was represented as milligrams of β-carotene equivalent per gram of dried TP. The extract was estimated in triplicate.

### High-performance liquid chromatography (HPLC) fingerprinting of TPE

An Agilent 1260 series was used for the HPLC analysis. Eclipse C18 column (4.6 mm × 250 mm i.d., 5 μm) was used for the separation. Water (A) and 0.05% trifluoroacetic acid in acetonitrile (B) were the components of the mobile phase, which had a flow rate of 0.9 ml/min. The linear gradient was sequentially programmed into the mobile phase as follows: 0 min (82% A); 0–5 min (80% A); 5–8 min (60% A); 8–12 min (60% A); 12–15 min (82% A); 15–16 min (82% A) and 16–20 (82% A). At 280 nm, the multi-wavelength detector was monitored. The freeze-dried TPE (100 mg) was re-dissolved in 1 ml HPLC grade methanol and filtrate through a 0.2 μm filter sterilized membrane before the injection. The sample solutions (5 μl) were injected into the HPLC device, the column was kept at a constant temperature (40 °C), then the retention times of the identified compounds of interest were measured. The extract was estimated in triplicate. The concentration of the sample is determined by comparing the peak area of the sample with that of the standard relative to the standard’s concentration. 18 standard (gallic acid, chlorogenic acid, catechin, methyl gallate, coffeic acid, syringic acid, pyrocatechol, rutin, coumaric acid, vanillin, ferulic acid, naringenin, daidzein, querectin, cinnamic acid, apigenin, kaempferol and hesperetin) were used.

### Assessment of TPE effect on the probiotic strain’s viability

The probiotic bacteria (*Lactobacillus acidophilus, Bifidobacterium bifidum* or their mixture) were enumerated using the pour plate technique and De-Man Rogoza and Sharp (MRS) agar media (Oxoid) according to De Man et al.^13^ in the presence of TPE (2%). The plates were incubated anaerobically at 37 °C for 48 h. The microbiological results were expressed as log colony-forming units (CFU).

### In silico predictions of TPE anti-inflammatory through docking assay

The binding affinity score of TPE bioactive molecules namely, ellagic acid and rutin on the active sites of the inflammatory cytokines namely, human IL-1β (PDB: 1HIB) and tumor necrosis factor (TNF-alpha) (PDB: 2AZ5)^14^ was determined using PyRx tool. 3D chemical structures of ellagic acid and rutin (SMILE code) were obtained from the PubChem database “https://pubchem.ncbi.nlm.nih.gov/” and drawn using the Chem Draw tool. The target proteins and ligands were loaded into the PyRx software and then converted to PDBQT^15^, the GRID parameters were maximized, then the docking assay was performed^16^. Complex of protein and ligand was visualized using PyMOl and Discovery Studio.

#### Preparation of the probiotics pellet

The probiotic bacteria were activated individually using De-Man Rogoza and Sharp (MRS) broth and incubated for 24 h at 37 °C anaerobically to obtain high biomass. The cell pellets were obtained by centrifugation at 5000 rpm, for 15 min at 4 °C. The obtained cell pellets were washed by a sterile saline solution (0.9% w/v of NaCl) and stored at 4 °C for the encapsulation procedure.

### Microencapsulation technique of probiotics and TPE

The sodium alginate (3% w/v) was dissolved in distilled water and stirred continuously until the fully dissolved. The sodium alginate solution was sterilized in an autoclave for 15 min at 121 °C. The TPE (15% on a dry basis) was swirled magnetically for 5 min. At 16,000 rpm, the mixture was homogenized for 2 min. To produce the co-encapsulated TPE with probiotics, Probiotic pellets at a concentration of around 8 log CFU/ml were added to the mixture of sodium alginate solution and TPE at a concentration of 25%. The mixture was then agitated using a magnetic stirrer at room temperature for 20 min to combine the cells thoroughly with the mixture. The extrusion technique^17^ was used to produce either the microcapsules of TPE or the co-encapsulated TPE with probiotics. With moderate stirring for 30 min, either the sodium alginate and TPE mixture or the sodium alginate, TPE, and probiotic strains mixture was separately extruded into the hardening solution (CaCl_2_, 0.2 M). Filtration was used to collect the produced microcapsules, which were subsequently cleaned using a sterile saline solution. The microcapsules were then placed in the refrigerator until use.

### Encapsulation efficiency (EE)

According to Toprakç et al.^17^, the encapsulation efficiency (EE) for either the microcapsules of TPE or the co-encapsulated TPE with probiotics was determined. EE was expressed by the following equation:

2 $${\text{EE }}\left( \% \right) \, = {\text{ TPC}} - {\text{SPC}}/{\text{TPC}}$$

where TPC is the total phenolic content and SPC is the surface phenolic content. Simply, 100 mg of the microcapsules were dissolved in 3 ml of a 50:8:42 (v/v/v) mixture of ethanol, acetic acid, and water. The mixture was exposed to an ultrasonic bath at ambient conditions for 1 min after mixing with a vortex, and TPC was then measured colorimetrically. SPC was assessed colorimetrically after dissolving of 100 mg of the microcapsules in a 3:1 ethanol-to-methanol solution by being placed in an ultrasonic bath for 5 min at room temperature.

### Scanning electron microscope (SEM) of TPE with probiotics microcapsules

The morphological shape of TPE and TPE with probiotic microcapsules were the same. Therefore, SEM was examined only for the co-encapsulated TPE with probiotics. To remove moisture content before the scanning, the microcapsules were dried in a lab oven at 40 °C for 2 h. After coating the microcapsules using Quorum Q 150 ES (United Kingdom) for 60 s with a gold layer of around 20 nm, a high-resolution scanning electron microscope model (TESCAN VEGA 3 with field emission gun, Czech Republic) was utilized to examine the morphological structure of these microcapsules.

### The animal experiment

#### Animals

Adult male albino rats of Wistar strain weighing 173.5 ± 6.10 g as Mean ± SD were used in this study and obtained from the Animal Care Unit of the National Research Centre, Cairo, Egypt. Animals were kept individually in stainless steel metabolic cages at temperature (23 ± 1 °C), relative humidity (55 ± 1%), and 12/12 h light/dark cycles. A maintenance standard diet was prepared according to Reeves et al.^18^ to contain 12% protein, 10% corn oil, 10% sucrose, 58.5% starch, 5% fiber, 3.5% AIN-93 salt mixture, and 1% AIN-93 vitamin mixture. Food and water were provided ad libitum.

#### Preparation of the jelly candy

Jelly candy was chosen as a suitable delivery vehicle for the soft-produced microcapsules. According to Halim et al.^19^, gelatin was dissolved in water to prepare the jelly candy. After heating to a boil and cooling to 40 °C, the microcapsules of TPE, or the microcapsules of TPE with probiotics, of the required concentrations, were added to the jelly solution, then the mixture was poured into mould and cooled for an hour in the refrigerator.

### Grouping and treatments

A total of thirty rats were acclimatized to laboratory conditions for 1 week before the starting time of the experiment, then divided into five groups (n = 6) with equal average weight as follows:

- Control normal group (NC): Untreated rats.
- Indomethacin group (INDO): Rats were subcutaneously injected with 10 mg/kg^20^ of indomethacin for 2 sequential days (16th and 17th).
- Jelly candy group (JC): Rats daily fed 10 g jelly candy (from the 1st to the 18th day) and injected with 10 mg/kg of indomethacin for 2 sequential days (16th and 17th).
- Jelly candy with the microcapsules of TPE group (JC + TPE): Rats daily fed 10 g jelly candy containing 0.8 g of TPE microcapsules (from the 1st to the 18th day) and injected with 10 mg/kg of indomethacin for 2 sequential days (16th and 17th).
- Jelly candy with the microcapsules of TPE and probiotics group (JC + TPE + PC): Rats daily fed 10 g jelly candy containing 1 g of co-encapsulated TPE and probiotics (from the 1st to the 18th day) and injected with 10 mg/kg of indomethacin for 2 sequential days (16th and 17th).

TPE microcapsules (0.8 g) and the co-encapsulated TPE with probiotics (1 g) were produced from the extrusion of an equal volume of sodium alginate solution. The co-encapsulated TPE and probiotics (1 g) contained 10^8^ CFU of probiotics. All over the experiment period, the animals fed on the aforementioned maintenance-balanced diet. The animal’s body weight was recorded at the baseline and the end of the experiment. Food intake was recorded daily. Body weight gain or loss was calculated. On the 18th day, blood was collected and the animals were scarified by cervical dislocation and dissected to remove the stomach, small intestine, and colon, which were then gently washed with saline. Both the colon and small intestine were measured for length. Through the microscopical examination, the scoring pattern method^21^ was applied to a section of rat stomach tissues and scored as follows:

**Table:** 

No. visible change	0
Hyperemia at sites	1
Lesions with a diameter of l mm or less	2
Lesions with a diameter of 2 mm or less than 5	5
Lesions with a diameter of more than 2 mm	6
Number 5–10	7
Number > 10	8

Freshly excised stomach and small intestine from each group were washed with saline, and preserved in a 10% formaldehyde solution for histopathological examination. The paraffin-embedded blocks were sectioned at 5-micron thickness and stained with Hematoxylin and Eosin^22^ for histopathological examination by a light microscope (Olympus BX50, Japan). The histopathological alterations of the stomach and intestine were graded^23^ as follows:

**Table:** 

No. changes	0
Mild changes	+
Moderate changes	+ +
Severe changes	+ + +

### Estimation of the oxidative and inflammatory markers in the intestine and stomach homogenates

Small portions of the stomach and intestine were individually used to prepare homogenates (10% w/v) in a cold homogenization buffer (100 mM potassium phosphate buffer, pH 7.4). The homogenates were centrifuged for 10 min at 4 °C, and supernatants were used for conducting the needed biochemical assays. According to Sedlak and Lindsay^24^, Nishikimi et al.^25^, Montgomery and Dymock^26^, Aebi^27^, and Ohkawa et al.^28^, the collected supernatants were tested for reduced glutathione oxidase (GSH), superoxide dismutase (SOD), nitric oxide (NO), catalase (CAT), and malondialdehyde (MDA), respectively using UVPC spectrophotometer (Jasco V-730, serial No. A 112,361,798, Japan). Sandwich ELISA detection kits (SinoGeneclon Biotech Co., Ltd.) were used to measure interleukin-6 (IL-6), tumor necrosis factor (TNF-α), and interleukin-1β (IL-1β).

### Estimation of serum liver and kidney biochemical indicators

According to Rheinhold and Seligron^29^, Bessey et al.^30^, Zimmerman and Weinstein^31^, and Reitman and Frankel^32^, respectively, the total protein, alkaline phosphatase (ALP), lactate dehydrogenase (LDH), aspartate transaminase (AST), and alanine transaminase (ALT) in each rat’s serum were estimated using a UVPC spectrophotometer (Jasco V-730, serial No. A 112,361,798, Japan). Larsen^33^, Fawcett and Scott^34^, and Doumas et al.^35^ methods were used to assess the amounts of creatinine, urea, and albumin, respectively using a UVPC spectrophotometer. The albumin/globulin (A/G) and globulin ratios were computed.

### Statistical analysis

Using SPSS version 21(SPSS Inc., Chicago, Illinois, USA), statistical analysis was conducted. The results were presented as mean ± standard error (SE) and one-way analysis of variance (ANOVA) was used to conduct a statistical analysis of the results. To examine the statistical variances between groups, the Duncan test was performed. *P* ≤ 0.05 was used to determine the difference’s statistical significance.

### Ethics approval and consent to participate

The study was given approval by the National Research Center’s Medical Research Ethics Committee (MREC) with Ethical Approval Certificate No 1496072023. and all procedures were carried out in accordance with the ethical standards, all methods are reported in accordance with ARRIVE guidelines. Experimental research and field studies on plants including the collection of plant material, comply with relevant institutional, national, and international guidelines and legislation.

## Results and discussion

### Characteristics of TPE

#### Yield, total phenolic content, radical scavenging activity and carotenoid content of TPE

Tomato pomace is one of the most valuable agro-industry by-products. This may be attributed to its enrichment of bioactive compounds such as polyphenols, carotenoids, pectin, dietary fibers, and fatty acids^35^. In this study, ultrasound-assisted extraction (UAE) was used in the preparation of TPE because it has been demonstrated to be an efficient method for obtaining bioactive molecules with high antioxidant activity from tomato seeds and peels. Additionally, it has the potential to be scaled up for the commercialization of tomato industrial wastes^9^. TPE recorded yield, total phenolic content, radical scavenging activity and carotenoids at 16.5 ± 0.04%, 2.27 ± 0.2 mg GAE, 4.7 ± 0.1 mg TE, and 184.7 ± 2.08 mg β-carotene equivalent per gram of dried TP, respectively (see Supplementary Table S1 online). The measured amounts are larger than those found in previous studies on tomato pomace from other varieties^10,36^. The obtained results are in accordance with the previous report^37^ on Egyptian tomato waste (skin and seeds) where tomato pomace was extracted by UAE with different solvents. El-Malah et al.^37^ found that aqueous and ethanol extracts of TP recorded the highest yield, antioxidant activity, and total phenolic content among the other solvent extracts. Additionally, it was found that the ethanol extract had a higher concentration of carotenoids than the aqueous extract^37^.

### HPLC phenolic profile of TPE

**Figure 1:**
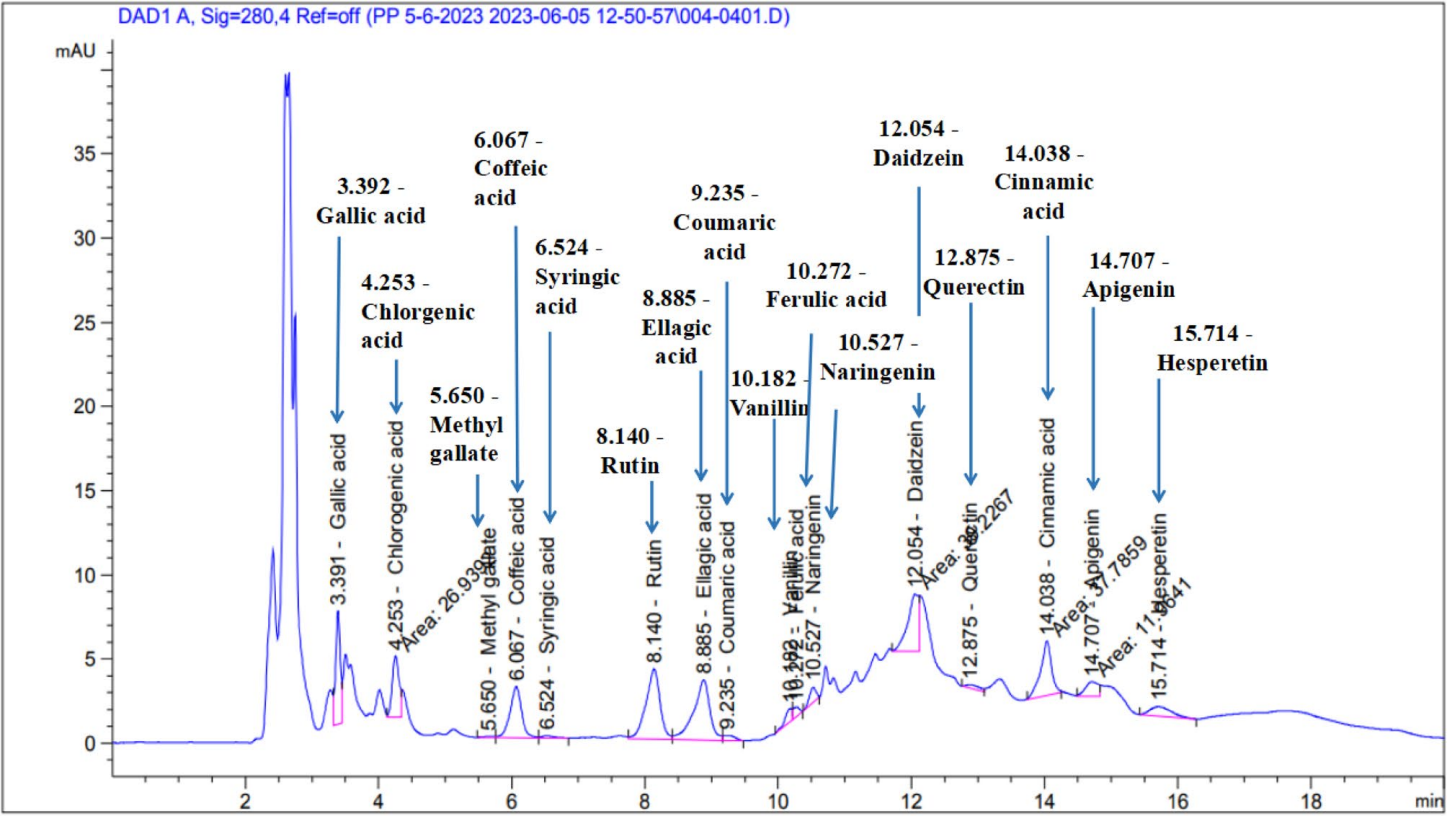
HPLC chromatogram of TPE’s phenolic compounds.

In this study, 16 phenolic compounds were identified in TPE by the HPLC. According to the findings (Table 1 and Fig. 1), the most abundant phenolic compounds in TPE were ellagic acid, rutin, chlorogenic acid, and gallic acid. El-Malah et al.^37^ reported that rutin and rutin derivatives were the identified polyphenols in the pomace of the same Egyptian tomato variety. In different tomato varieties, chlorogenic acid, rutin, and naringenin were the main identified phenolic compounds in the extract of tomato seeds^20^ whereas Grassino et al^38^ found that chlorogenic acid and its derivatives were the major identified compounds in the extract of tomato skin.

**Table 1:** HPLC phenolic profile of TPE.

	Conc. (µg/g dried TP)
Gallic acid	143.79 ± 0.03
Chlorogenic acid	184.45 ± 0.03
Methyl gallate	2.46 ± 0.02
Coffeic acid	139.67 ± 0.04
Syringic acid	5.90 ± 0.00
Rutin	355.71 ± 0.03
Ellagic acid	563.28 ± 0.02
Coumaric acid	5.82 ± 0.01
Vanillin	12.09 ± 0.01
Ferulic acid	12.88 ± 0.01
Naringenin	36.47 ± 0.02
Daidzein	121.17 ± 0.03
Querectin	21.89 ± 0.02
Cinnamic acid	34.80 ± 0.02
Apigenin	45.54 ± 0.01
Hesperetin	36.54 ± 0.02

### Effect of TPE on the probiotic’s viability

With the TPE, the counts of *Lactobacillus acidophilus* and *Bifidobacterium bifidum* or their mixture (see Supplementary Table S2 and Fig. S1A–C, respectively online) increased from 22 × 10^5^, 28 × 10^5^, and 33 × 10^5^ CFU, respectively (the initial counts) to 18 × 10^8^, 25 × 10^8^, and 45 × 10^8^ CFU, respectively. These findings indicate to the promotion effect of TPE on the viability of *L. acidophilus* and *B. bifidum* or their mixture. The beneficial effect of TPE on the viability of probiotic bacteria may be attributed to its antioxidant activity and polyphenol content (as noted in the obtained results) that act as prebiotics. The beneficial effects of polyphenols on the viability of probiotic bacteria were demonstrated in several studies as confirmed by Ibrahim et al.^39^ who found that polyphenols of pomegranate peel improved the viability of probiotic bacteria. According to Garca-Alonso et al.^40^, there was a positive correlation between polyphenols intake and Lactobacillus bacteria while the polyphenols intake negatively correlated to the presence of Enterobacteriaceae. Garca-Alonso et al.^40^ also declared that the regulation of bacterial populations and SCFA concentrations may be positively affected by polyphenol ingestion.

### Docking assay

Inhibitors of tumor necrosis factor are among the conventional treatments of gastrointestinal inflammation^6^. The inhibitors of tumor necrosis factor bond to its active sites, preventing receptor binding and thereby stopping the activation of downstream signaling complexes that induce inflammation and other signaling pathways^41^. Therefore, human TNF-α and IL-1β inflammatory cytokines were chosen as the target proteins. Enrichment of TPE in ellagic acid and rutin stimulated us to carry out the docking assay between them and the target proteins, however further studies on the other compounds are required. According to the findings (Table 2), rutin interacted with the active sites of TNF-α with hydrophobic interactions at the TYR 59, HIS 15, VAL 123 and LEU 55, LEU 57, and LEU 157 residues. Ellagic acid interacted with the active sites of TNF-α with hydrophobic interaction at the TYR 59 residue. Rutin interacted with the active sites of IL-1β with 3 conventional hydrogen bonds and hydrophobic interaction at the LYS 77 residue. Ellagic acid interacted with the active sites of IL-1β with 2 conventional hydrogen bonds and hydrophobic interaction at the PHE 133 residue. Zia et al.^41^ investigated the TNF-α inhibition activity of several compounds in silico and suggested that the docked compounds with strong hydrophobic interactions and hydrogen bond contacts with TNF-α inhibited the inflammation.

**Table 2:** Docking of rutin and ellagic acid with TNF-α and IL-1β.

Target cytokines protein	Ligand	Binding affinity (kcal/mol)	The 3D map of the binding	2D Protein–ligand interactions
TNF-α (2AZ5)	Rutin	− 7.4		
	Ellagic acid	− 8.1		
IL-1β (1HIB)	Rutin	− 7.5		
	Ellagic acid	− 7.1		

### Characteristics of the produced microcapsules

#### Encapsulation efficiency (EE)

Despite of the potential health benefits of the natural phenolic compounds and antioxidants, the instability of these compounds’ physical and chemical properties represent a challenge when they are used in industrial processes. These limitations are overcome by microencapsulation^42^. The encapsulation techniques promote the viability and the effective delivery of living cells to the intended location. Furthermore, the encapsulation techniques protect living cells from the challenging physiological conditions of the stomach^43^. In this context, co-extrusion encapsulation of TPE and probiotics was used in this study. Encapsulation efficiency of TPE and TPE with probiotics were at 88.2 ± 0.8 and 90.2 ± 1.04%, respectively. According to Al-Hindi and Abd El Ghani^44^, probiotic bacterial counts increased upon adding polyphenols from pomegranate peel to fermented milk beverages. Using an aqueous delivery system, Shinde et al.^45^ showed that co-extrusion encapsulation of probiotic *L. acidophilus* and apple skin polyphenol extract recorded microencapsulation efficacy more than 96%. In the study by Al-Moghazy et al.^46^, co-extrusion encapsulation of *B. lactis* probiotic with pomegranate peel extract recorded a microencapsulation efficiency of more than 89.65%.

#### Morphological structure by SEM

**Figure 2:**
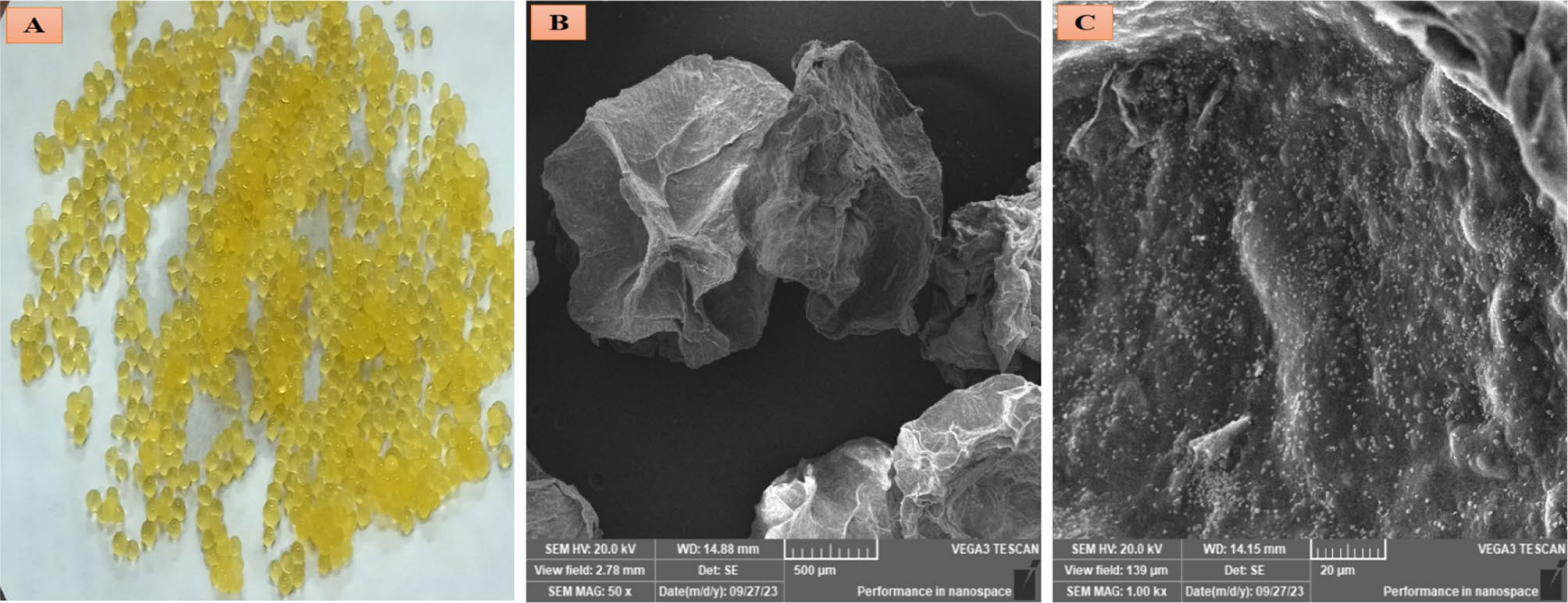
Photograph and SEM images of TPE with probiotic microcapsules. (**A**): photoghraph of the TPE with probiotic microcapsules (Magnification 4×), (**B**): SEM image of the whole microcapsules (Magnification 50×), (**C**): SEM image of a cross-section of the microcapsule structure (Magnification 1k×).

The homogeneous shape of TPE and probiotic encapsulated beads is observed from the photograph image (Fig. 2A). The beads’ particles recorded about 1.5 mm in diameter. The spherical surface of the TPE and probiotic encapsulated beads could be seen in the SEM images (Fig. 2B,C). In the SEM image, the shrinkage on the surface of the beads’ particles might be attributed to the drying process.

### The protective effect of the produced microcapsules against indomethacin-induced enterocolitis

#### Effect of the produced microcapsules on the growth performance

Non-steroidal anti-inflammatory drugs (NSAIDs) such as aspirin and indomethacin are commonly used to treat pain, fever, and inflammation. However, administration of NSAIDs, due to its widespread use and ease of accessibility, causes gastrointestinal tract (GIT) ulcers. Indomethacin promotes the development of ulcers by suppressing prostaglandin production and increasing the production of free oxygen radicals, which in turn stimulate various of transcription factors resulting in the differential expression of some genes involved in inflammatory pathways^47^. According to Shanmugam et al.^20^, the treatment of indomethacin (10 mg/kg) was fatal and caused perforating tiny intestinal ulcers in rats through 2–4 days. In the current study, rats were injected with indomethacin for 2 sequential days (16th and 17th) to induce GIT ulceration. The protective effect of microencapsulated TPE either alone or with probiotics against GIT ulceration was evaluated. *L. acidophilus* and *B. bifidum* probiotic bacteria were selected in this study as they are the most common probiotics reported to have beneficial health effects. Additionally, they have been used safely in several food products^48^. In accordance with Shanmugam et al.^20^, upon the treatment with indomethacin, the body weight gain (see Supplementary Table S3 online) of the INDO group (34.67 g) significantly was less than the NC group (40.50 g). Also, the length of both the small intestine and colon of the INDO group (102.5 and 16.17 cm, respectively) significantly was higher than the NC group (95.42 and 13.83 cm, respectively). In contrast, the body weight gain was higher in rats with either TPE or the co-encapsulated TPE with probiotics than the INDO group. While, the length of both the small intestine and colon was less in rats treated with either TPE or the co-encapsulated TPE with probiotic than the INDO group as strong evidence for the reduction of inflammation condition. The results revealed that the jelly candy was not involved in any improvement in the studied parameters or improvement in inflammation, but it was only a way to deliver the produced microcapsules.

### Effect of the produced microcapsules on oxidative and inflammatory markers in the stomach and intestine homogenates

**Figure 3:**
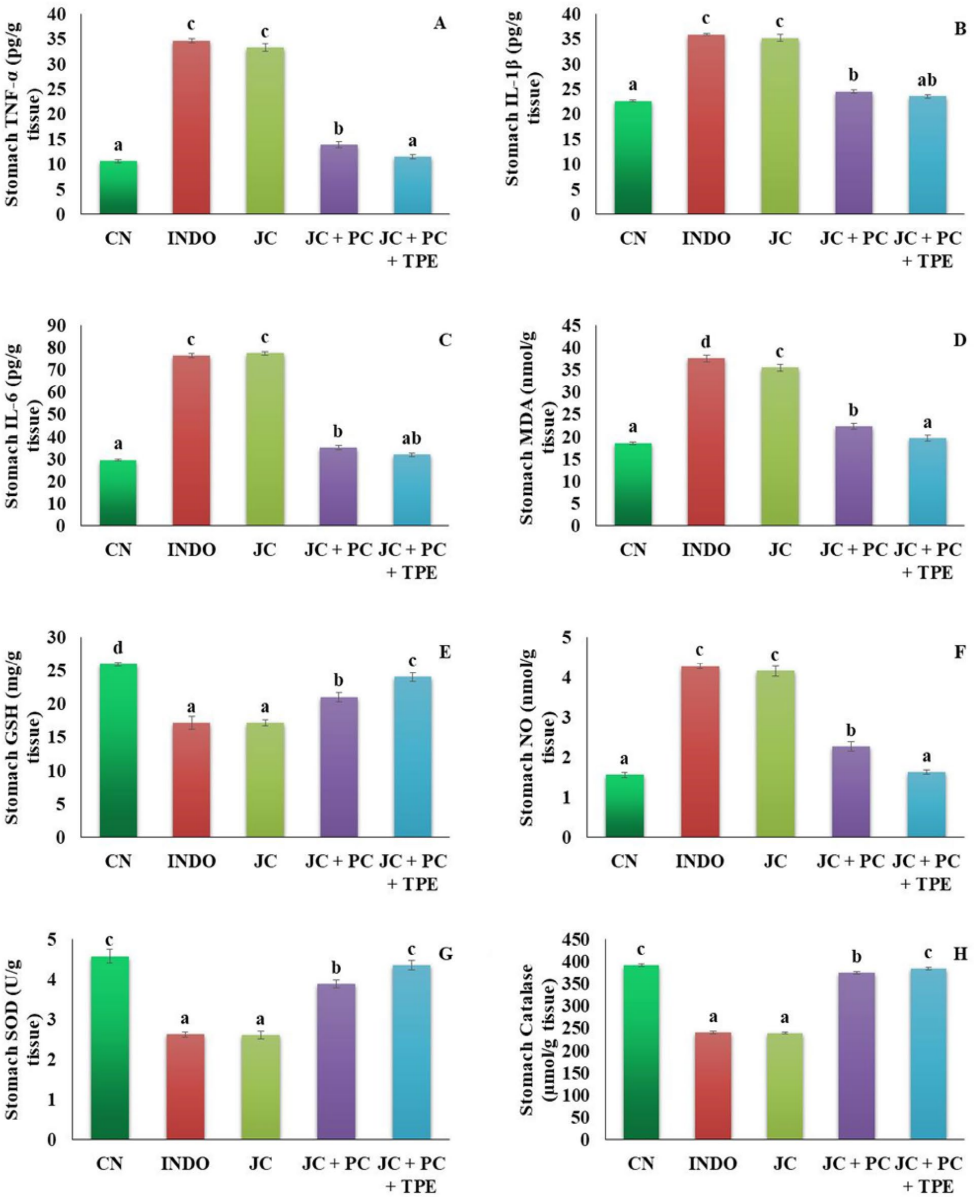
Effect of the produced microcapsules on stomach inflammatory and oxidative markers. (**A**): TNF-α, (**B**): IL-1β, (**C**): IL-6, (**D**): MDA, (**E**): GSH, (**F**): NO, (**G**): SOD, (**H**): CAT. NC: normal control group, INDO: indomethacin group, JC: jelly candy group, JC + TPE: rats treated with jelly containing the microcapsules of tomato pomace extract, JC + TPE + PC: rats treated with jelly containing the microcapsules of tomato pomace extract with probiotics. Data are mean values ± SE (n = 6). A significant difference at *P* ≤ 0.05 can be identified from a different superscript letter in on the bars.

**Figure 4:**
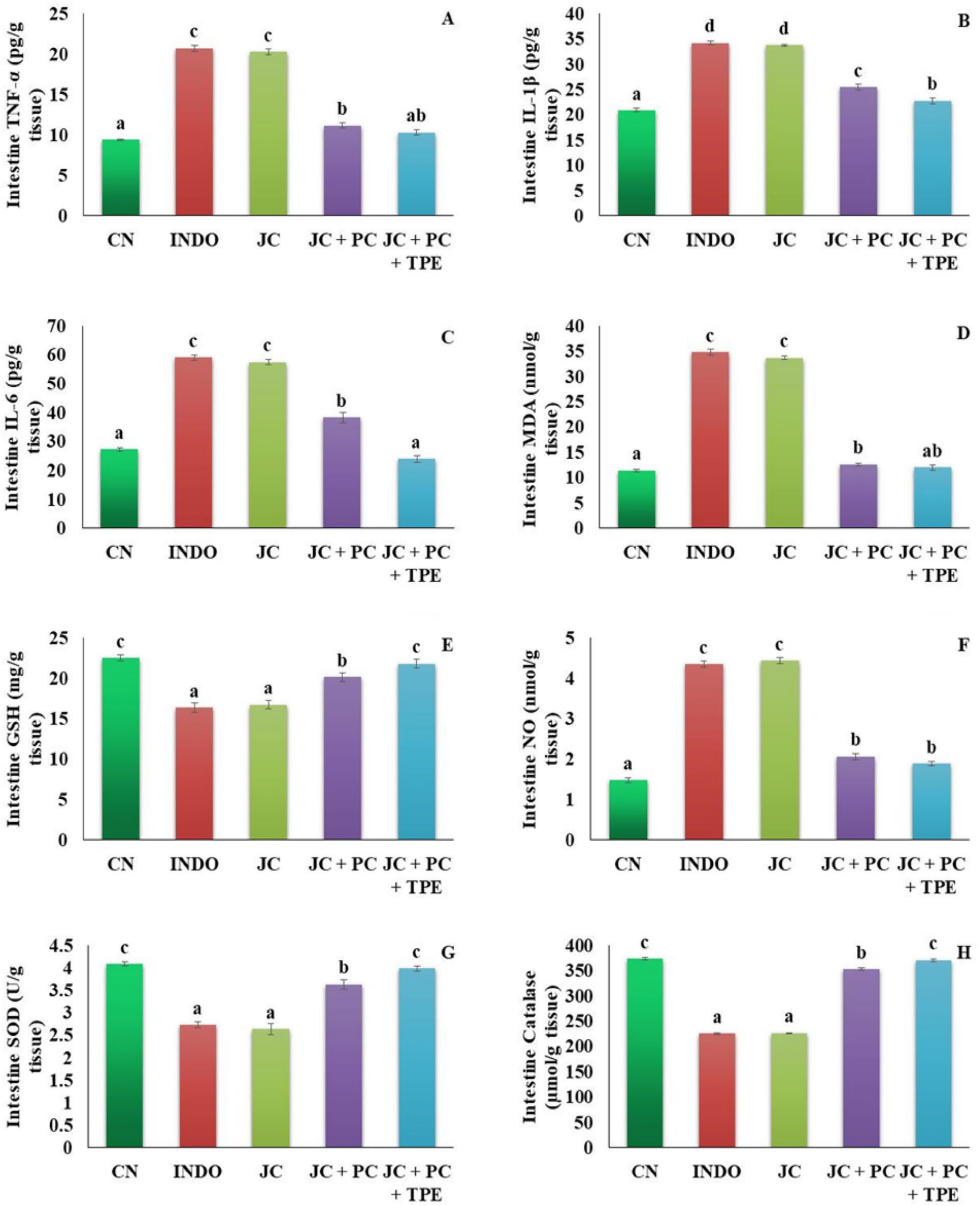
Effect of the produced microcapsules on intestinal inflammatory and oxidative markers. (**A**): TNF-α, (**B**): IL-1β, (**C**): IL-6, (**D**): MDA, (**E**): GSH, (F): NO, (**G**): SOD, (**H**): CAT. NC: normal control group, INDO: indomethacin group, JC: jelly candy group, JC + TPE: rats treated with jelly containing the microcapsules of tomato pomace extract, JC + TPE + PC: rats treated with jelly containing the microcapsules of tomato pomace extract with probiotics. Data are mean values ± SE (n = 6). A significant difference at *P* ≤ 0.05 can be identified from a different superscript letter on the bars.

The pathophysiology of GIT ulceration and the excessive generation of ROS are both known to be significantly influenced by oxidative stress and an imbalance between oxidants and antioxidants^49^. Figures 3 and 4 show the findings of the oxidative and inflammatory markers of the stomach and intestine. In accordance with the results reported by Shanmugam et al.^20^ and Danisman et al.^5^, indomethacin treatment significantly altered the levels of inflammatory markers in the stomach and intestine, including IL-6 (76.33 ± 0.95 and 58.93 ± 0.93 pg/g tissue, respectively), IL-1β (35.84 ± 0.16 and 34.17 ± 0.31 pg/g tissue, respectively) and TNF-α (34.65 ± 0.46 and 20.72 ± 0.37 pg/g tissue, respectively) when compared to the normal control group. The levels of stomach and intestine oxidative markers including SOD (2.62 ± 0.07 and 2.73 ± 0.07 U/g tissue, respectively), CAT (239.83 ± 2.98 and 224.67 ± 1.14 μmol/g tissue, respectively), GSH (17.17 ± 10.94 and 16.32 ± 0.6 mg/g tissue, respectively), NO (4.27 ± 0.07 4.35 ± 0.07 nmol/g tissue, respectively) and MDA (37.5 ± 0.76 and 34.78 ± 0.57 μmol/g tissue, respectively) were drastically altered in the INDO group comparing to the normal control group. On the other hand, rats treated with TPE or the co-encapsulated TPE with probiotics exhibited lower levels of the stomach and intestine inflammatory markers as well as MDA and NO than the INDO group. Furthermore, rats treated with TPE or the co-encapsulated TPE with probiotics showed significantly higher activities of the antioxidant enzymes SOD, CAT and GSH than the INDO group. Comparable to the results of the INDO group, rats treated with the co-encapsulated TPE with probiotics exhibited significant improvement than TPE microcapsules. These effects might be attributed to the combined effect of probiotics and the bioactive compounds of TPE. According to the findings (see Supplementary Table S1 online and Table 1), carotenoids, polyphenols, and flavonoids are among the major bioactive compounds of TPE. Carotenoids (β-carotene, lutein, and lycopene) have antioxidant and anti-inflammatory properties, making them potentially beneficial phytochemicals for gut health^50^. Polyphenols can transfer electrons to the free radicals and suppress them, preventing cell damage. Additionally, polyphenols activate the antioxidant enzymes and reduce oxidative stress and inflammation^51^. The anti-inflammatory effect of flavonoid derivatives (EGCG, rutin, apigenin, naringenin) and phenolic acids was demonstrated by several reports^52^. Probiotics have become more well-liked in recent years as a result of their ability to treat diseases caused by inflammation. Probiotics are implicated in the enhancement of intestinal permeability, regulation of immune function, and reduction of pro-inflammatory cytokines^53^. Additionally, it has been demonstrated that probiotic bacteria and/or bacterial metabolites affect the host by changing the amounts of both endogenous and exogenous ROS resulting in oxidative stress reduction^54^.

### Effect of the produced microcapsules on liver and kidney biochemical indicators

Supplementary Table S4 online includes information on the results of liver and kidney functions for the different groups. In line with Mahmoud et al.^55^ and Stephanie et al.^56^ but in contrast to the findings of Shanmugam et al.^20^, the levels of serum ALP (129.33 U/L), AST (43 U/L), ALT (33.17 U/L) and LDH (282.17 U/L) increased in the INDO group. However, no significant change among the groups was recorded for urea, creatinine, albumin, and total protein. Rats treated with TPE and the co-encapsulated TPE with probiotics recorded liver and kidney functions that were closely comparable to those of the control group. High serum activity of AST, ALT, and ALP enzymes, may be useful quantitative markers for assessing GIT injury. These enzymes are normally not present in serum; but, when tissue damage occurs, some of these enzymes may leak into the serum^57^. Similarly, the combination of probiotics and TPE bioactive substances may play a role in the significant effect on liver and kidney functions. Previously, *L. acidophilus* and *B. bifidum* administration in conjunction with aflatoxin maintained liver and kidney functions close to the normal rats^58^^.^ Phenolic and flavonoid compounds, including ellagic acid and rutin, demonstrated protective effects against liver injury and preserved liver functions^59,60^.

### Effect of the produced microcapsules on macroscopic changes and ulceration score

The macroscopic examination revealed that indomethacin had a more severe impact on the stomach than the small intestine, where it manifested as chronic intestinal inflammation. Therefore, the stomach ulcer score was determined. The calculated ulcer score (see Supplementary Table S5 online) was significantly less in TPE and the co-encapsulated TPE with probiotic groups compared with the INDO group. Moreover, the protection % of the co-encapsulated TPE with probiotics (85.91%) was significantly higher than the TPE microcapsules (71.94%). These results verified the protective effect of TPE and the co-encapsulated TPE with probiotic against gastrointestinal tract (GIT) ulceration. The decrease in ulcer score and the increase in the protection % of TPE may be attributed to the phenolic compounds as confirmed by^20^. Furthermore, the combination action of TPE and probiotics may be responsible for the decrease in lesion scores in rats treated with the co-encapsulated TPE and probiotics.

### Effect of the produced microcapsules on morphological and histopathological alterations of stomach and intestine

**Figure 5:**
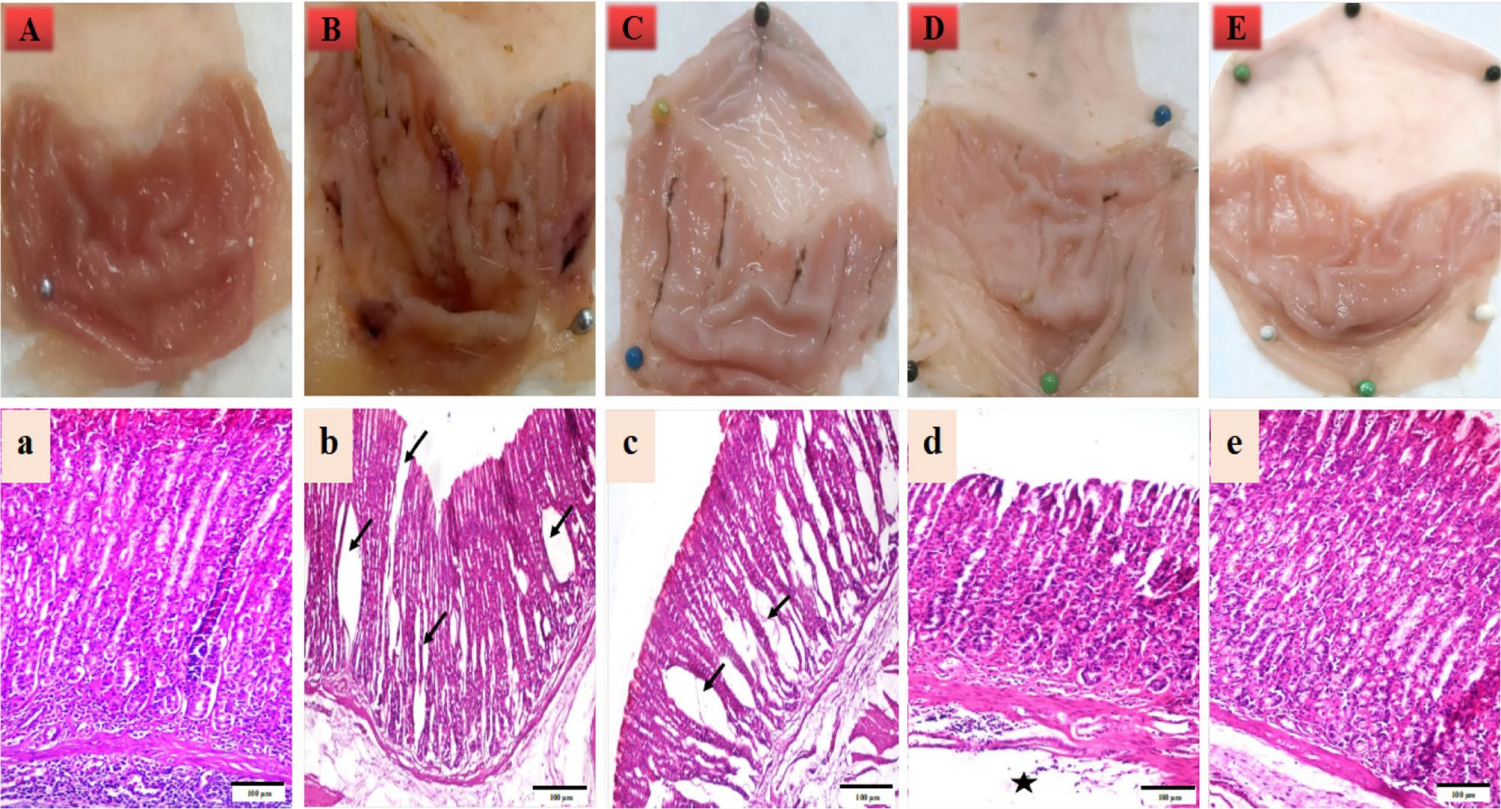
Photographs (× 4) and light micrographs of stomachs from the studied groups (stained with H&E X100). (**A**) normal control, (**B**) indomethacin group, (**C**) jelly candy group, (**D**) TPE microcapsules group, E: TPE and probiotics microcapsules group. (**a**) Normal control group (0), (**b**) indomethacin group with multiple mucosal erosions (arrows) and submucosal edema together with blood vessel congestion (+ + +), (**c**) jelly candy group with multiple mucosal erosions (arrows) and submucosal edema without blood vessel congestion (+ +), (**d**) TPE microcapsules group with normal mucosa and mild submucosal edema (star) ( +), (**e**) TPE and probiotic microcapsules group with intact mucosa and normal submucosa (0).

All the recorded lesions in the stomach and intestine were scored as shown in Supplementary Table S6 online. The control animals’ stomach tissues had typical gross morphology (Fig. 5A). The gastric mucosa of the indomethacin-treated rats displayed obvious ulceration and bleeding foci (Fig. 5B). The macroscopic examination of the stomach from the jelly candy group displayed obvious ulceration and bleeding foci (Fig. 5C). The macroscopic examination of the stomach from the TPE microcapsules group showed mild erosion in the gastric mucosa (Fig. 5D). The macroscopic examination of the stomach from the the co-encapsulated TPE with probiotics group showed normal gastric mucosa (Fig. 5E).

Histopathologically, the stomach from the normal control group showed normal mucosa with normal intact epithelium and normal submucosa without edema nor blood vessel congestion (Fig. 5a). The stomach from the indomethacin group revealed multiple mucosal erosions and submucosal edema together with blood vessels congestion (Fig. 5b). Jelly candy group showed as multiple mucosal erosions, as the indomethacin group, together with submucosal edema without blood vessel congestion (Fig. 5c). In TPE microcapsules group, the stomach mucosa was as normal intact as recorded in control group, while the submucosa showed mild edema (Fig. 5d), while the stomach from the co-encapsulated TPE with probiotics group recorded no histological alterations than the control normal group (Fig. 5e).

**Figure 6:**
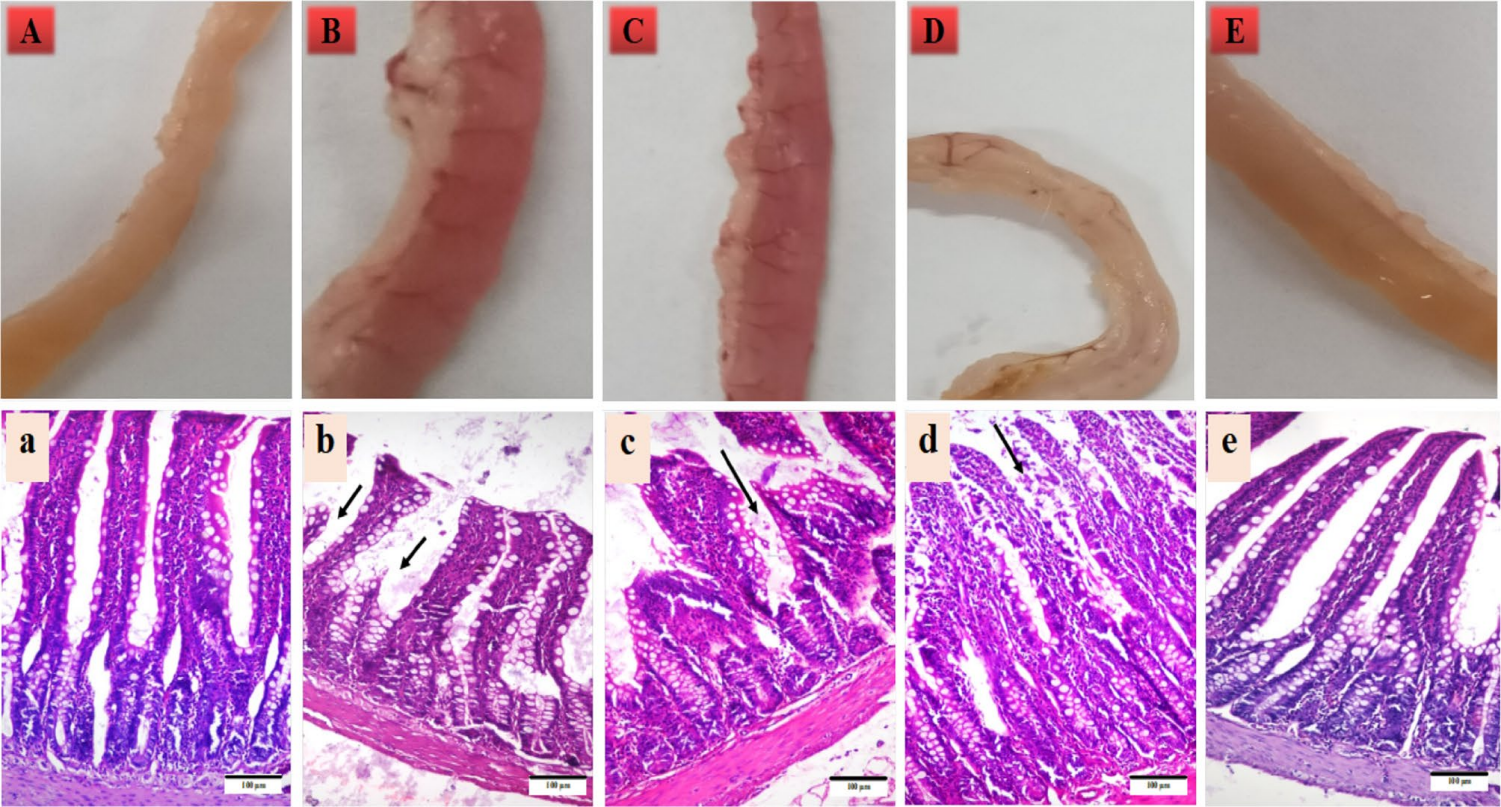
Photographs (× 4) and light micrographs of intestines from the studied groups (stained with H&E X100). (**A**) normal control, (**B**) indomethacin group, (**C**) jelly candy group, (**D**) TPE microcapsules group, E: TPE and probiotics microcapsules group. (**a**) Normal control group (0), (**b**). indomethacin group with multiple mucosal erosions (arrows) and submucosal edema (+ + +), (**c**) jelly candy group with few mucosal erosions (arrows), there is no submucosal edema (+ +), (**d**) TPE microcapsules group with mild mucosal epithelium sloughing and normal submucosa ( +), (**e**) TPE and probiotic microcapsules group with intact mucosa and normal submucosa (0).

The control animals’ intestinal tissues had typical gross morphology (Fig. 6A). The indomethacin-treated animals’ intestines displayed chronic inflammation (Fig. 6B). The intestine from the jelly candy group displayed chronic inflammation also (Fig. 6C). The macroscopic examination of the intestine from the TPE microcapsules group showed a normal intestine (Fig. 6D). The macroscopic examination of the intestine from the co-encapsulated TPE with probiotics group showed a normal intestine (Fig. 6E).

Histopathologically, the intestine from the normal control group showed normal mucosa with normal intact epithelium, intestinal glands, and normal submucosa without edema nor blood vessel congestion (Fig. 6a). The intestine from the indomethacin group revealed multiple mucosal erosions and submucosal edema (Fig. 6b). The jelly candy group showed fewer mucosal erosions than the indomethacin group, there was no submucosal edema (Fig. 6c). In the TPE group, the intestinal mucosa revealed mild epithelial sloughing, there was no edema in the submucosa (Fig. 6d), while the intestine from the co-encapsulated TPE with probiotics group recorded no histological alterations than the control normal group (Fig. 6e).

## Conclusions

The obtained results demonstrated the antioxidant activity of tomato pomace extract, which may be strongly related to its phenolic component and carotenoid content. The presence of TPE in the growth media increased the viability of *Lactobacillus acidophilus* and *Bifidobacterium bifidum* probiotic bacteria. According to the docking results, the extract’s highest concentrations of phenolic compounds, ellagic acid, and rutin, were demonstrated to bind to the active sites of TNF-α and IL-1β cytokines, suggesting that these compounds may be responsible for the extract’s anti-inflammatory actions. When probiotic bacteria were co-encapsulated with TPE, high encapsulation efficiency was recorded. The reduction in oxidative stress and inflammation was demonstrated by the reduction in stomach and intestine MDA, NO, IL-1β, IL-6, and TNF-α levels and the increase in CAT, SOD, and GSH activities in rats treated with TPE microcapsules and the co-encapsulated TPE with probiotics. The produced microcapsules are thought to be promising candidates for protecting against indomethacin-induced erosion and stomach ulcers.

### Supplementary Information

Supplementary Information.

## Data Availability

All data generated or analyzed during this study are included in this published article.

## Abbreviations

TP: Tomato pomace
TPE: Tomato pomace extract
HPLC: High-performance liquid chromatography
SEM: Scanning electron microscope
EE: Encapsulation efficiency
JC: Jelly candy
IBD: Inflammatory bowel disease
IL-1β: Interleukin 1 beta
TNF-α: Tumor necrosis factor alpha
IL-6: Interleukin 6
DPPH: 1,1-Diphenyl-2-picrylhydrazyl
ALP: Alkaline phosphatase
AST: Aspartate transaminase
ALT: Alanine transaminase
LDH: Lactate dehydrogenase
GIT: Gasrointestinal tract
CAT: Catalase
GSH: Reduced glutathione
NO: Nitric oxide
SOD: Superoxide dismutase
MDA: Malondialdehyde
ROS: Reactive oxygen species
