# Supplementary material for: Protective effect of tomato pomace extract encapsulated in combination with probiotics against indomethacin induced enterocolitis
Source: Sci Rep. 2024 Jan 27;14:2275. doi: 10.1038/s41598-024-52642-y (PMC10821949; doi:10.1038/s41598-024-52642-y)
Supplement: Supplementary file 1 — Supplementary Information. [file 41598_2024_52642_MOESM1_ESM.doc]

**Protective effect of tomato pomace extract encapsulated in combination with probiotics against indomethacin induced eneterocolitis**

**Karem Fouda^1^, Ahmed M. Mabrouk^2^, Sherein S. Abdelgayed^3^, Rasha S. Mohamed^1^***

^1^ Nutrition and Food Sciences Department, National Research Centre, Dokki, Cairo, Egypt.

^2^ Dairy Department, National Research Centre, Dokki, Cairo, Egypt.

^3^ Pathology Department, Faculty of Veterinary Medicine, Cairo University, Cairo, Egypt.

**Table 1. The yield, total phenolic content, radical scavenging activity and total carotenoids of TPE**

| **Yield (%)** | 16.5 ± 0.04 |
| --- | --- |
| **Total phenolic content (mg GAE/ g dried TP)** | 2.27 ± 0.2 |
| **Radical scavenging activity (mg TE/ g dried TP)** | 4.7 ± 0.1 |
| **Total carotenoids (mg β-carotene eq/ g of dried TP)** | 184.7 ± 2.08 |

**Table 2.** The effect of TPE on the probiotics viability

|  | **Initial count (CFU)** | **Count after incubation (CFU)** |
| --- | --- | --- |
| ***Lactobacillus acidophilus*** | 22 x 10^5^ | 18 x 10^8^ |
| ***Bifidobacterium bifidum*** | 28 x 10^5^ | 25 x 10^8^ |
| ***L. acidophilus* and *B. bifidum* mixture** | 33 x 10^5^ | 45 x 10^8^ |

**Table 3. Effect of the produced microcapsules on body and food parameters**

|  | **NC** | **INDO** | **JC** | **JC + TPE** | **JC +**  **TPE + PC** |
| --- | --- | --- | --- | --- | --- |
| **Initial Body Weight (g)** | 173.67^a^±2.65 | 173.33^a^±2.73 | 173.83^a^±2.88 | 173.50^a^±2.40 | 173.33^a^±2.72 |
| **Final Body Weight (g)** | 214.17^a^±3.15 | 208.00^a^±2.66 | 207.33^a^±4.06 | 213.33^a^±2.74 | 213.50^a^±2.72 |
| **Body Weight Gain (g)** | 40.50^b^±1.06 | 34.67^a^±1.14 | 33.50^a^±2.51 | 39.83^b^±1.05 | 40.17^b^±0.70 |
| **Total Food Intake (g)** | 310.33^a^±2.88 | 308.33^a^±2.74 | 304.33^a^±2.62 | 311.83^a^±3.18 | 308.00^a^±3.78 |
| **Food Efficiency Ratio** | 0.13^b^±0.01 | 0.11^a^±0.01 | 0.11^a^±0.01 | 0.13^b^±0.01 | 0.13^b^±0.01 |
| **Small Intestine Length (cm)** | 95.42^a^±1.36 | 102.50^b^±1.33 | 102.17^b^±1.47 | 96.33^a^±1.11 | 95.17^a^±1.66 |
| **Colon Length (cm)** | 13.83^a^±0.59 | 16.17^b^±0.38 | 15.75^b^±0.65 | 13.60^a^±0.60 | 13.50^a^±0.62 |

NC: normal control group, INDO: indomethacin group, JC: jelly candy group, JC+TPE: rats treated with jelly containing the microcapsules of tomato pomace extract, JC+TPE+PC: rats treated with jelly containing the microcapsules of tomato pomace extract with probiotics. Data are mean values ± SE (n = 6). A significant difference at P ≤ 0.05 can be identified from a different superscript letter in the same row.

**Table 4. Effect of the produced microcapsules on liver and kidney indicators**

|  | **NC** | **INDO** | **JC** | **JC + TPE** | **JC +**  **TPE + PC** |
| --- | --- | --- | --- | --- | --- |
| **AST (U/l)** | 36.83^a^±1.08 | 43.00^b^±1.03 | 43.83^b^±0.60 | 37.67^a^±0.67 | 36.83^a^±0.60 |
| **ALT (U/l)** | 29.50^a^±0.85 | 33.17^c^±0.79 | 32.83^bc^±0.79 | 30.67^ab^±1.08 | 28.67^a^±0.42 |
| **ALP (U/l)** | 124.67^ab^±1.26 | 129.33^b^±2.60 | 126.00^ab^±1.12 | 125.68^ab^±0.75 | 124.33^a^±1.36 |
| **LDH (U/l)** | 256.83^a^±2.81 | 282.17^c^±1.89 | 275.67^b^±1.93 | 271.33^b^±2.18 | 259.33^a^±1.63 |
| 1. **Protein (g/dl)** | 7.25^a^±0.13 | 7.03^a^±0.21 | 7.10^a^±0.22 | 7.35^a^±0.13 | 7.42^a^±0.15 |
| **Albumin (g/dl)** | 4.62^a^±0.20 | 4.30^a^±0.21 | 4.25^a^±0.18 | 4.45^a^±0.15 | 4.57^a^±0.10 |
| **Globulin (g/dl)** | 2.63^a^±0.26 | 2.73^a^±0.20 | 2.85^a^±0.28 | 2.90^a^±0.26 | 2.85^a^±0.15 |
| **A/G ratio** | 1.90^a^±0.30 | 1.64^a^±0.18 | 1.61^a^±0.25 | 1.62^a^±0.20 | 1.63^a^±0.10 |
| **Urea (mg/dl)** | 27.97^a^±0.32 | 28.83^a^±0.95 | 28.23^a^±0.31 | 28.08^a^±0.76 | 27.53^a^±0.50 |
| **Creatinine (mg/dl)** | 0.37^a^±0.01 | 0.37^a^±0.01 | 0.39^a^±0.02 | 0.38^a^±0.02 | 0.36^a^±0.01 |

NC: normal control group, INDO: indomethacin group, JC: jelly candy group, JC+TPE: rats treated with jelly containing the microcapsules of tomato pomace extract, JC+TPE+PC: rats treated with jelly containing the microcapsules of tomato pomace extract with probiotics. Data are mean values ± SE (n = 6). A significant difference at P ≤ 0.05 can be identified from a different superscript letter in the same row.

**Table 5. Effect of the produced microcapsules on stomach ulcer score**

|  | **Score** | **Affected area (%)** | **Protection (%)** |
| --- | --- | --- | --- |
| **NC** | - | - | - |
| **INDO** | 6.50^c^±0.18 | 81.25^b^±2.28 | - |
| **JC** | 6.58^c^±0.20 | 82.30^b^±2.50 | 0 |
| **JC + TPE** | 1.83^b^±0.28 | 7.83^a^±0.98 | 71.94±4.21 |
| **JC + TPE + PC** | 0.93^a^±0.33 | 4.66^a^±1.38 | 85.91±4.94 |

NC: normal control group, INDO: indomethacin group, JC: jelly candy group, JC+TPE: rats treated with jelly containing the microcapsules of tomato pomace extract, JC+TPE+PC: rats treated with jelly containing the microcapsules of tomato pomace extract with probiotics. Data are mean values ± SE (n = 6). A significant difference at P ≤ 0.05 can be identified from a different superscript letter in the same column.

**Table 6.** Histopathological lesion scoring of stomach and intestine

|  | | **Grades of the lesions** | | | |
| --- | --- | --- | --- | --- | --- |
|  |  | **0**  **(Negative)** | **+**  **(Mild)** | **++**  **(Moderate)** | **+++**  **(Sever)** |
| **NC** | **Stomach** | √ |  |  |  |
|  | **Intestine** | √ |  |  |  |
| **INDO** | **Stomach** |  |  |  | √ |
|  | **Intestine** |  |  |  | √ |
| **JC** | **Stomach** |  |  | √ |  |
|  | **Intestine** |  |  | √ |  |
| **JC + TPE** | **Stomach** |  | √ |  |  |
|  | **Intestine** |  | √ |  |  |
| **JC +**  **TPE + PC** | **Stomach** | √ |  |  |  |
|  | **Intestine** | √ |  |  |  |

NC: normal control group, INDO: indomethacin group, JC: jelly candy group, JC+TPE: rats-treated with jelly containing the microcapsules of tomato pomace extract, JC+TPE+PC: rats-treated with jelly containing the microcapsules of tomato pomace extract with probiotics.


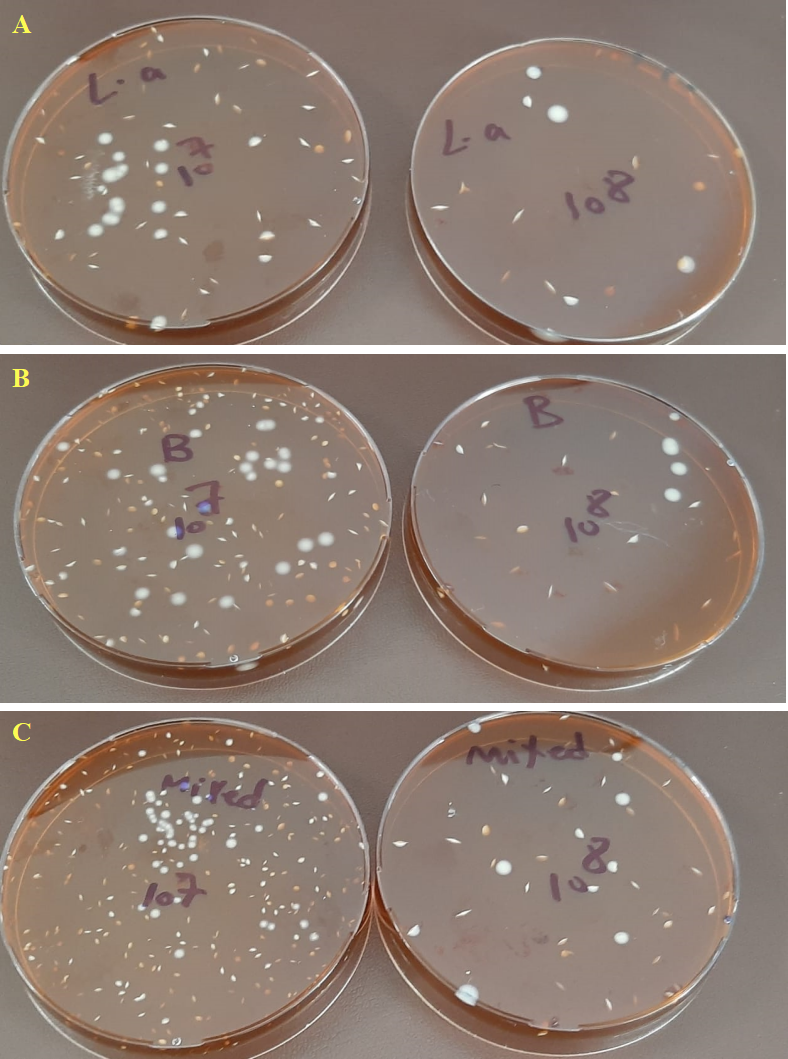


**Figure 1.** Effect of TPE on the counts of *Lactobacillus acidophilus* (A) and *Bifidobacterium bifidum* (B) and their mixture (C).
